# Frequency and intensity of facilitation reveal opposing patterns along a stress gradient

**DOI:** 10.1002/ece3.3855

**Published:** 2018-01-22

**Authors:** Laure Gallien, Damaris Zurell, Niklaus E. Zimmermann

**Affiliations:** ^1^ Swiss Federal Research Institute WSL Birmensdorf Switzerland; ^2^ Centre for Invasion Biology Department of Botany and Zoology Stellenbosch University Matieland South Africa

**Keywords:** asymmetric facilitation, coexistence, commensalism, co‐occurrence patterns, mutualism, stress gradient

## Abstract

Disentangling the different processes structuring ecological communities is a long‐standing challenge. In species‐rich ecosystems, most emphasis has so far been given to environmental filtering and competition processes, while facilitative interactions between species remain insufficiently studied. Here, we propose an analysis framework that not only allows for identifying pairs of facilitating and facilitated species, but also estimates the strength of facilitation and its variation along environmental gradients. Our framework combines the analysis of both co‐occurrence and co‐abundance patterns using a moving window approach along environmental gradients to control for potentially confounding effects of environmental filtering in the co‐abundance analysis. We first validate our new approach against community assembly simulations, and exemplify its potential on a large 1,134 plant community plots dataset. Our results generally show that facilitation intensity was strongest under cold stress, whereas the proportion of facilitating and facilitated species was higher under drought stress. Moreover, the functional distance between individual facilitated species and their facilitating species significantly changed along the temperature–moisture gradient, and seemed to influence facilitation intensity, although no general positive or general negative trend was discernible among species. The main advantages of our robust framework are as follows: It enables detecting facilitating and facilitated species in species‐rich systems, and it allows identifying the directionality and intensity of facilitation in species pairs as well as its variation across long environmental gradients. It thus opens numerous opportunities for incorporating functional (and phylogenetic) information in the analysis of facilitation patterns. Our case study indicated high complexity in facilitative interactions across the stress gradient and revealed new evidence that facilitation, similarly to competition, can operate between functionally similar and dissimilar species. Extending the analyses to other taxa and ecosystems will foster our understanding how complex interspecific interactions promote biodiversity.

## 1 INTRODUCTION

As the rise of biogeography, researchers have sought to understand how plant–plant interactions change along environmental gradients, and what consequences this has for the composition of plant communities (e.g., von Humboldt & Bonpland, 1807). Two types of interactions are dominant in shaping community composition: competition and facilitation (Brooker & Callaghan, 1998). Competitive or facilitative interactions can be defined as interactions in which the presence of one species alters the environment (or occupies space) in a way that reduces or enhances growth, survival, and reproduction of a second species (Bronstein, 2009; Craine, Fargione, & Sugita, 2005; McIntire & Fajardo, 2014). The relative importance of these two processes has been shown to vary along environmental gradients, with competition generally dominating in communities of low‐abiotic stress, while facilitation increases in importance with abiotic stress (framed in the *stress gradient hypothesis*; Bertness & Callaway, 1994; Callaway & Walker, 1997; Choler, Michalet, & Callaway, 2001; Callaway et al., 2002; Michalet, Schöb, Lortie, Brooker, & Callaway, 2014).

Previous work on facilitative interactions has repeatedly demonstrated that facilitation can act as a major force structuring plant communities, and helped identifying its putative underlying mechanisms (McIntire & Fajardo, 2014). Nonetheless, our understanding of this process remains limited. On the one hand, even though facilitation is usually thought to be more important under stressful conditions (Callaway, 2007), it may not necessarily be restricted to stressful conditions only (Holmgren & Scheffer, 2010; McIntire & Fajardo, 2014). Indeed, it may happen that only few keystone species provide important facilitative services to many facilitated species under stressful conditions, while under less stressful conditions facilitation may be of lower intensity but provided by a larger number of species. Such a situation may explain why signals of facilitation are often lost under environmental conditions that are favorable to plant growth. Therefore, to be able to capture the full extent of facilitative interactions, we need to develop a community‐level understanding of how facilitation varies, both in intensity and frequency, along large environmental stress gradients. Key components of such a community‐level assessment should include both, the identification of each facilitating–facilitated species pair in the communities, and the estimation of the degree to which these facilitative interactions contribute to the increase in fitness of facilitated species (hereafter called *facilitation intensity*; Welden & Slauson, 1986).

On the other hand, the mechanisms determining the nature and magnitude of facilitation remain poorly understood (McIntire & Fajardo, 2014; Schöb, Butterfield, & Pugnaire, 2012). Facilitation mechanisms can be symmetric or asymmetric, and involve direct or indirect drivers. Asymmetric facilitation indicates that one species (the benefactor or facilitating species) will disproportionately favor another species (the beneficiary or facilitated species) more than it can mutually profit from this species. For instance, tall plants may protect shorter plants from ultraviolet radiations (*asymmetric facilitation* or commensalism, as shorter plants do not protect taller plants from radiations), while species with similar flower color may attract the same pollinators (*symmetric facilitation* or mutualism; Brooker & Callaghan, 1998; Chu et al., 2009; Lin, Berger, Grimm, & Ji, 2012). Within a functional framework, if species facilitate each other via the same mechanism (e.g., pollinator attraction via similar flower color, or soil stabilization via root reinforcement) then the intensity of symmetric facilitation should increase with species functional similarities, whereas that of asymmetric facilitation should increase with functional dissimilarities (Butterfield & Briggs, 2011; Cavieres & Badano, 2009; Gross et al., 2009). However, if species facilitate each other via different mechanisms (such as direct and indirect effects of the benefactor on the local abiotic or biotic environment; see McIntire & Fajardo, 2014 for a list of examples) it remains unclear how species functional similarities are expected to relate to their facilitation intensity. Therefore, a key challenge today is to quantify the relationship between facilitation (a)symmetry, intensity, and species functional (dis)similarities.

These knowledge gaps—about the relationship between facilitation (a)symmetry, intensity, and species functional (dis)similarities—are not due to a lack of experiments or observational studies, but for the large part rather due to a lack of methodological approaches allowing for investigations of large environmental gradients and of species‐rich communities, where multispecies interactions are not known a priori and where indirect interactions may be frequent (such as intransitive competition; Gallien, 2017; Gallien, Zimmermann, Levine, & Adler, 2017). Indeed, most studies on facilitation mechanisms to date have relied on: (1) the comparison of communities in paired plots containing or not the facilitating species (e.g., species growing inside versus outside of a cushion plant; Butterfield et al., 2013); (2) experiments testing the effect of removing the facilitating species (e.g., Albrecht et al., 2015; Callaway et al., 2002; Cipriotti & Aguiar, 2015; Michalet et al., 2015); or (3) monitoring long‐term changes in community composition during primary succession (e.g., Martorell & Freckleton, 2014). These approaches are all valuable, yet they strongly rely on a priori knowledge about the facilitating species and/or extensive monitoring efforts. There is, thus, a strong need for screening methods based on comparably simple data, which allow for analyzing multispecies interaction links without experiments.

Here, we propose and apply a simple but robust framework for exploring facilitation patterns without a priori information on the local species and the processes that drive species co‐occurrences, and without need for experimental manipulation. This screening procedure allows for identifying pairwise facilitative interactions in species‐rich communities and for tracking their variation along large environmental gradients. We use the output of this approach to specifically investigate the relationships between facilitation intensity and species functional (dis)similarities along a long stress gradient using a large community dataset. This helps us progressing toward a better understanding of the facilitation process in plant communities and toward designing more complex and targeted experiments.

We first describe our proposed approach and evaluate its performance using a community assembly simulation model (VirtualCom; Münkemüller & Gallien, 2015). As these simulations show that our approach works well and facilitation is accurately detected, we are confident to apply it on a large dataset of 1,134 plant community plots from the Zermatt region (Switzerland) and tackle key questions related to facilitative interactions. Specifically, we asked the following: (1) How do facilitation frequency and intensity change along environmental gradients? (2) Does the functional distance between the facilitating and facilitated species change along environmental gradients? (3) Is facilitation intensity influenced by the functional similarity between the involved species? Finally, we discuss future avenues and potential research questions that can be answered using our approach.

## 2 MATERIALS AND METHODS

### 2.1 The facilitation screening procedure

We propose a screening framework that elaborates on the widely studied co‐occurrence patterns (e.g., Boulangeat, Gravel, & Thuiller, 2012; Diamond, 1975; Jackson, Somers, & Harvey, 1989; López, Valdivia, Rivera, & Rios, 2013; Ulrich & Gotelli, 2007), and thus, only requires community relevés with recordings of the relative cover of coexisting species (i.e., at a relatively small grain size at which species interact). By combining co‐occurrence analyses with analyses of co‐abundance, we aim at detecting facilitation for species pairs within a specific environment. Our method estimates for each pair of co‐occurring species A and B, whether species A facilitates species B and by which intensity.

**Figure 1:**
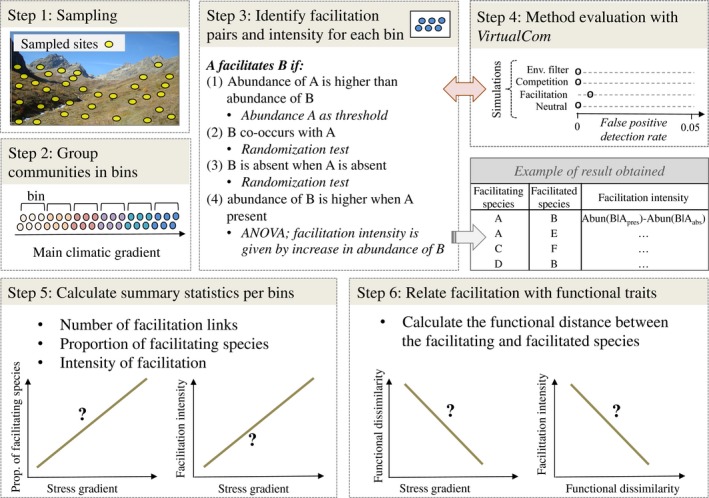
The six major steps of the proposed analysis framework. Once the community relevés have been sampled (step 1), the main environmental gradient(s) among them shall be identified (for instance with a principal component analysis), and then the communities are grouped into “bins” of similar environmental conditions (step 2). Next, for each bin, all possible species pairs are tested for facilitative interactions (see also Table 1), and the facilitation intensity is estimated as the difference in abundance of the facilitated species when the facilitating species is present versus absent (step 3). The performance of the applied methodology is then evaluated on artificial communities simulated with different assembly rules using the *VirtualCom* simulation model (step 4). After this preliminary test, we calculate a number of facilitation metrics within each bin (such as the number of facilitation links or the average facilitation intensity received by the facilitated species), and analyze how they change along the stress gradients (for instance with regression models; step 5). Finally, using functional trait information one can test for each pair of species the relationship between the facilitation intensity, the functional distance, and the environmental gradient (step 6)

To avoid confusion with environmental filtering signals, our method groups community relevés into ecologically very narrow bins of similar environmental conditions (Figure 1, steps 2). Within each bin, we then identify facilitating–facilitated species pairs by testing all possible species pairs (Figure 1, steps 3). For each species pair A and B, species A is considered as facilitating species B if it fulfills the following four requirements. (1) The relative cover of A is higher than the relative cover of B, meaning that we assume a facilitator to have higher plant cover than the facilitated species, (2) B co‐occurs with A more often than expected at random, and (3) B is more often absent when A is absent than expected at random. These two latter requirements were tested for significance via randomization tests where species B occurrences were permutated among communities independently of the presence of species A, with 499 randomizations per species (using a .025 significance threshold). Finally, (4) the relative cover of B is significantly higher in community relevés where A is present than when A is absent (Table 1). This was assessed using ANOVA tests. When significant, the amount of increase in relative cover of species B (when A is present vs. absent) was used as an estimate of facilitation intensity received by B (Figure 1, step 3). In other words, this framework allows for testing whether a facilitated species benefits from a facilitator more than can be expected by chance, both regarding its presence and its abundance: The presence of the facilitating species A increases both the likelihood of occurrence and the abundance of the species B, while A is not necessarily affected by B (Table 1 and Figure 1 step 3). Note that constraining the relative cover of the facilitator species to be higher than the one of the facilitated species generally brings a stronger focus on asymmetric facilitation patterns (e.g., facilitation via shading), but this constrain could be loosened to integrate symmetric facilitation (e.g., facilitation via pollinator attraction).

**Table 1:** Summary of the rules applied to identify facilitative interactions and how they can disentangle facilitation relative to other coexistence mechanisms

	Coexistence mechanism			
Identification rules	Facilitation	Environmental filtering	Competition	Neutral coexistence
The abundance of A is higher than that of B?	✓	✓/✗	✓/✗	✓/✗
A and B co‐occur more than by chance?	✓	✓	✗	✗
A is absent when B is absent?	✓	✓	✗	✗
A is more abundant when B is present?	✓	✗	✗	✗

Green ticks indicate significant positive responses to the identification rules, while red crosses indicate significant negative responses (ticks and crosses are represented together when both responses are possible).

Our method relies on two fundamental assumptions: (1) The environmental heterogeneity among the considered communities is negligibly small, and (2) the within site microhabitat heterogeneity is negligible. Indeed, if environmental heterogeneity is too high, fine‐scale environmental filtering processes may lead to differences in co‐occurrence and co‐abundance patterns similar to those expected from facilitation (i.e., if the niche of the species B is nested within the one of A and A's abundance is generally higher than that of B). We note that such assumption about environmental homogeneity is similarly made (although not always explicitly) in most analyses of community functional similarity patterns (e.g., when inferring environmental filtering and competition processes; Münkemüller et al., 2014; Willis et al., 2010). Additionally, our estimation of facilitation intensity relies on the assumption that an increase in relative cover of species is associated with an increase in its fitness. Although this assumption is likely to be verified in most situations, some systems might present exceptions that would preclude the utilization of our methodology.

### 2.2 Method validation with processed‐based community assembly simulations

As proof of concept that our approach is capable of reliably detecting facilitation and that it does not confound facilitation with other coexistence mechanisms (e.g., environmental filtering, competitive interactions, and neutral mechanisms), we used a virtual ecologist approach (Gallien, Carboni, & Münkemüller, 2014; Zurell et al., 2010), and compared four different simulation scenarios: facilitation, environmental filtering, competitive interactions, and neutral coexistence. To do so, co‐occurrence patterns were generated using the recently published community assembly model *VirtualCom* (Münkemüller & Gallien, 2015). *VirtualCom* has originally been developed to simulate community assembly under three possible processes, namely: environmental filtering, competitive interactions, and neutral coexistence. Here, we extended it to include the option of simulating facilitative interactions between pairs of species, where the probability of recruiting new individuals for the facilitated species increases with the abundance of the facilitating species (see Appendix S1 for detailed information on algorithms and simulations).

Additional to the three coexistence mechanisms mentioned above, we tested the usefulness of our approach in four different facilitation scenarios where the facilitating species facilitated either: 1, 2, 5, or 10 species. For each scenario (4 of facilitation + 3 of other mechanisms), we generated 50 different species pools containing 50 species each, where facilitating and facilitated species were chosen at random. From each species pool, we assembled 50 communities (with a carrying capacity of 200 individuals), which were then used as “sampled community data.” In each sampled community dataset, we evaluated whether each pair of species fulfilled the four requirements described above (see also Figure 1 step 3), in order to identify facilitating–facilitated species pairs and their associated facilitation intensity. Hence, this allowed us to test seven different scenarios, with 50 independent repetitions per scenario (7 scenarios × 50 repetitions × 50 community per repetition = 17,500 communities overall). For each repetition, we assessed the false‐positive (proportion of pairs identified as facilitating while they were not) and false‐negative (proportion of “true” facilitating pairs not identified by our method) error rates.

### 2.3 Method application with the Zermatt dataset

We then used this new screening method to detect and quantify facilitation in empirical data, using phytosociological relevés of ca. 2 m × 2 m in the Zermatt mountain region in Switzerland, composed of 1,242 plots sampled in natural and seminatural vegetation during the 1990s by several persons and summarized in Steiner (2002). The sampling covers an elevation gradient ranging from 1,536 m to 3,390 m a.s.l. (Appendix S1: Figure S1). When cleaning the dataset, we identified 108 sites containing species typical of very wet habitats indicating local water sources independent of climatic humidity gradients. We removed them from the dataset to avoid potential confounding effects of mixing different habitat types and microhabitat heterogeneity (which left us with 1,134 sites). Overall, the dataset contained a total of 574 species. Within each community plot, species relative cover was recorded using the Braun‐Blanquet cover scheme (see Appendix S1 for more details). In order to avoid statistical errors due to low sample size, we chose to work with those species that were present in at least 20 community plots, which left us with 262 species for further analyses (representing 87% of the vegetation cover on average).

### 2.4 Sampling along environmental gradients

**Figure 2:**
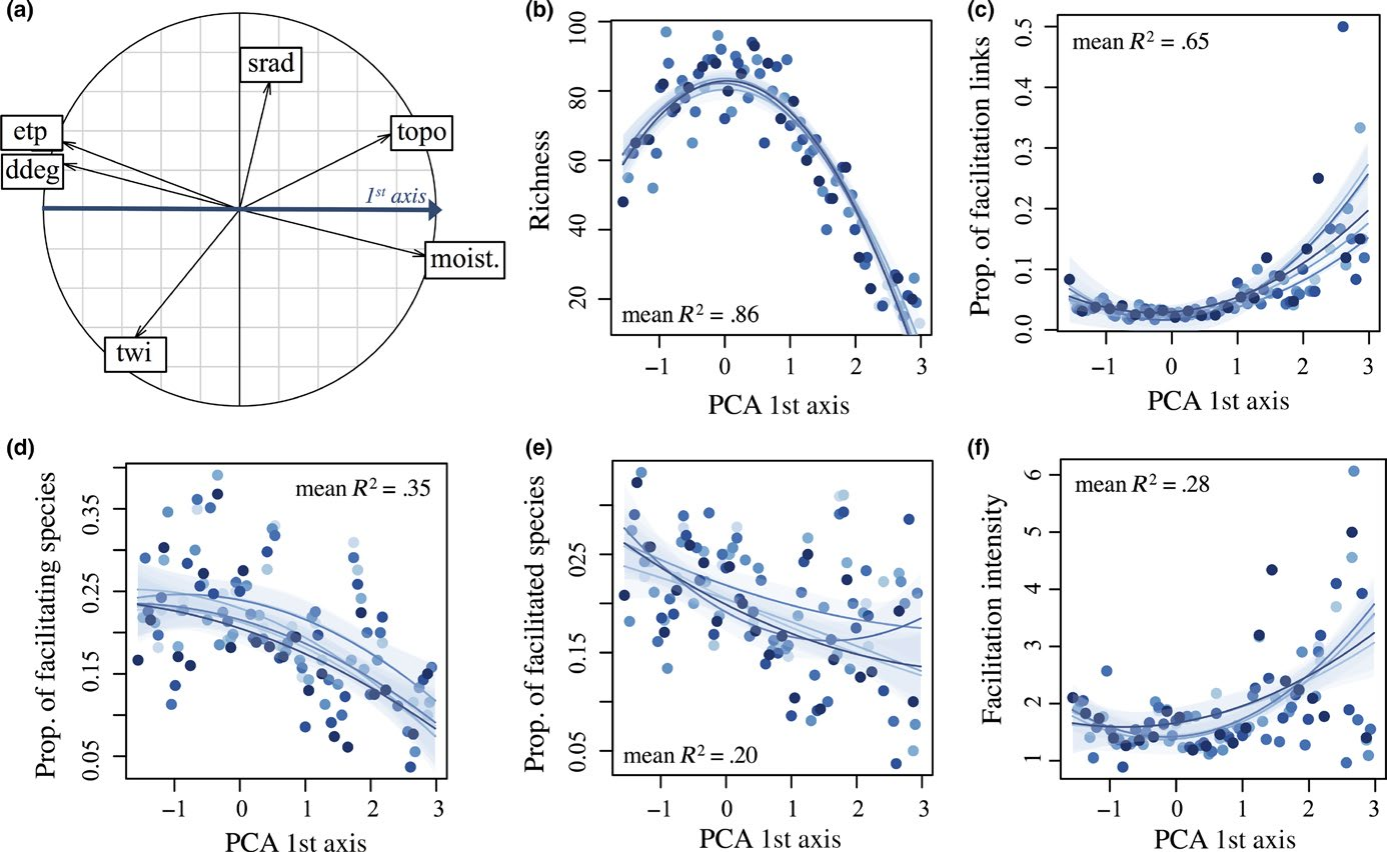
General trends in facilitation along the main environmental gradient of the study area. (a) The PCA 1st plan shows how the different environmental variables are related to the 1st PCA axis. Changes in community richness (b) and facilitation patterns (c‐f) along a warm/dry‐to‐cold/wet gradient. Facilitation measures, within each community, include the following: the proportion of facilitation links (c), the proportion of facilitating (d) and facilitated species (e), and the average received facilitation intensity (f). Seven different starting points were used for defining the bins (a bin is a set of communities encompassing similar environmental conditions) and are represented by seven different intensities of blue. Each dot represents the observed values, the solid lines the regression model (if significant), and the light blue shadings indicate the confidence intervals around the model fitted values

If co‐occurrence patterns are estimated across communities encompassing different environmental conditions, then facilitation may be confounded with environmental filtering. Indeed, two species may coexist more frequently than expected by chance only because they have similar ecological requirements, thus respond similarly to environmental filtering. To avoid such confusions, we calculated our co‐occurrence/co‐abundance measures within bins containing community plots with very similar environmental conditions. This step is also important for tracking changes in facilitation intensity along environmental gradients. We, thus, first performed a principal component analysis (PCA; using the R package *ade4*; Dray & Dufour 2007) on six topo‐climatic variables relevant for our studied region: (1) The mean annual potential evapotranspiration (etp) calculated based on the TURC formula (Turc 1963), (2) the annual mean moisture index (mind) calculated as the difference between precipitation and potential evapotranspiration, (3) the annual sum of degree‐days with a 0°C threshold (ddeg), (4) the annual sum of potential global solar radiation, (5) the site topography position (positive values indicating ridges and peak positions while negative values indicate gullies and valleys), and (6) the topographic wetness index (following Beven & Kirkby, 1979). These variables are considered to have direct physiological effects on species distributions and were used in many previous studies successfully (e.g., Randin et al., 2006; Zimmermann & Kienast, 1999). All variables were available at a 25 m spatial resolution, which is of fine enough grain to match the 2 × 2 m resolution of the community plots. The uncertainty in the temperature and precipitation data is summarized in Zimmermann and Kienast (1999), and is small enough to not confound the results along this steep and climatically very long gradient. We then chose the first PCA axis as representative of the stress gradient among sites for all further analyses because it revealed a warm/dry‐to‐cold/wet gradient (representing 57% of the intersite environmental differences, Figure 2a). Note that we used indicator species to remove sites that indicated local water sources independent of the climatic humidity gradient as described above.

Next, we grouped our 1,134 communities into bins of similar environmental conditions (according to the first PCA axis), with a bin breadth of 0.2 (a breadth identified as providing the most homogeneous number of communities across bins). For all further analyses, we considered only the bins containing at least 15 communities (39.1 communities on average), covering 978 sites in total and splitting the gradient into 26 bins in total. In order to evaluate the effect of the bin borders, we repeated the binning process seven times starting at different positions along the environmental gradient (but keeping the same bin breadth). Within each bin we only considered statistically relevant facilitation interactions if both the facilitating and the facilitated species were present in at least five communities.

### 2.5 Species‐level functional traits

To investigate differences in functional similarity between the facilitating and facilitated species along the studied environmental gradient and with changing facilitation intensity, we used six species‐specific, functional traits. These traits relate to the species’ microhabitat preferences and life history strategies (available in Flora Indicativa; Landolt, 2010), and thus to facilitation. The traits related to microhabitat preferences included species preferences for light availability, soil moisture level, humus level, and soil aeration; traits related to species life history strategy included species average leaf life span and CRS life strategies as defined by Grime (2006). For 10 species, these traits were not available, and therefore, all functional analyses were run on 252 instead of 262 species.

### 2.6 Statistical analyses

To answer our initial three questions, we followed three consecutive analytical steps. First, we tested for general trends in the frequency and intensity of facilitation along the environmental gradient (the PCA 1st axis). Second, we characterized the level of environmental stress (hereafter called *environmental filtering*) along the environmental gradient using functional diversity indices (Figure 1, step 5). This allowed us to then quantify the changes in functional dissimilarity among the facilitating (and among facilitated) species along the stress gradient. Third, for each facilitated species, we investigated whether: (1) the facilitation intensity received changed along the gradient, (2) the functional distance to the facilitators changed along the gradient, and (3) whether the facilitation intensity received by a facilitating species could be related to the functional distance to its facilitators (Figure 1, step 6).

#### 2.6.1 General trends along the environmental gradient

For each environmental bin, we estimated the species richness and four facilitation indices based on the identified facilitating–facilitated species pairs: (1) the proportion of facilitating species, (2) the proportion of facilitated species, (3) the proportion of facilitation links, and (4) the average facilitation intensity received by the facilitated species (defined as the mean increase in relative cover of all facilitated species when their facilitating species is present vs. absent). Each bin's position along the gradient (i.e., the PCA's 1st axis) was estimated as the mean position of all communities it contained. Next, we tested for significant relationships between these indices and environment using generalized linear models (GLMs) with linear and/or quadratic relationships and a stepwise, AIC‐based variable selection (Figure 1, step 5). The entire procedure was repeated for each of the seven different bin border placements considered.

#### 2.6.2 Functional trends along the environmental gradients

In order to estimate the intensity of environmental stress along our warm/dry‐to‐cold/wet gradient (PCA axis 1), we calculated the mean functional distance (MFD) between all pairs of species within each community from our set of six traits (using the Gower distance that can handle both continuous and categorical variables; Gower, 1971). Thereby, we expected that the stronger the environmental stress the more functionally similar is the coexisting species (compared to the full set of species in the dataset), as they should have similar traits to cope with the environmentally stressful conditions (Weiher & Keddy, 1999). We used the MFD standardized effect size (hereafter called MFD_SES_) to estimate the strength of this environmental filter in each bin. MFD_SES_ was obtained from null models by randomizing the functional distances among species, and thus by controlling for the community richness (999 repetitions). MFD_SES_ varies between 0 (perfectly similar species) and 1 (completely dissimilar species; details in Appendix S1). We then tested whether the MFD_SES_ scores changed along the environmental gradient using GLMs with linear and/or quadratic relationship followed by stepwise AIC variable selections.

Analogous to the procedure outlined above, we calculated functional similarity among all facilitating species and among all facilitated species, respectively, and evaluated whether and how facilitation mechanisms changed along the environmental gradient (MFD_Faciliting_, and MFD_Facilitated_, respectively). On the one hand, if facilitation is driven by one major mechanism (e.g., shading by tall plants or soil reinforcement by large root systems), we expect that all facilitating species tend to be functionally similar to each other (and all facilitated species tend to be similar to each other). On the other hand, if facilitation mechanisms change among facilitated species, we expect that facilitating species tend to be functionally dissimilar to each other (and all facilitated species tend to be dissimilar too). For this test, we compared the observed functional distance between the facilitating or facilitated species within a bin to the functional distance between any species within the bin. By means of a GLM, we tested whether MFD_Faciliting_ and MFD_Facilitated_ significantly varied along our warm/dry‐to‐cold/wet gradient (with linear and/or quadratic relationships and a stepwise, AIC‐based variable selection).

#### 2.6.3 Linking facilitation intensity with functional information

At the species level, we further investigated (1) whether the facilitation intensity received by each facilitated species varied along the environmental gradient, (2) whether the functional distance between each facilitated species and its facilitators changed along the gradient, and (3) whether the facilitation intensity received by these species can be explained by their mean functional distance to facilitators. For each species that was identified as being facilitated at least 10 times (i.e., in 10 different bins; that is 46 species in total), we tested these three types of relationships with GLMs including linear terms only (due to the limited number of observations) selected based on model AIC scores (Figure 1 step 5).

## 3 RESULTS

### 3.1 A new approach to detect facilitative interactions from community data

Our proposed approach to detect pairs of facilitating and facilitated species revealed great performance in tests using simulated data. First, our approach did not detect any facilitating interactions when there were none; that is when we simulated community assembly with scenarios of: environmental filtering (rate of false positive = 0), competition (rate of false positive = 0), and neutral coexistence (rate of false positive = 0). Second, under facilitation scenarios, we could identify the correct facilitating–facilitated species pairs, although the rates of false positives (i.e., species were wrongly identified as facilitating or facilitated while they were not) and false negatives (i.e., facilitating or facilitated species were not detected) were not null (Appendix S1: Figure S2). However, the false‐negative error rates were generally low (mean error rates < 0.05 for all scenarios) and decreased when the simulated number of facilitated species increased. The false‐positive error rates were very low (error rates < 0.01 for all scenarios), although they slightly increased when the number of facilitated species increased (Appendix S1: Figure S2). Overall, these error rates indicate that our test is generally conservative, especially when many species are facilitated in the studied system, meaning that some true facilitation pairs may be overlooked, but the probability of falsely identifying a species as facilitating is less than 1%. Thus, our approach is able to correctly identify facilitating and facilitated species pairs, given that the underlying assumptions are met (i.e., the environmental heterogeneity within the bins and within the communities is negligible).

### 3.2 Facilitation increases with environmental severity

Community species richness showed a unimodal response along the environmental gradient and was significantly higher at intermediate position of this gradient (Figure 2b). Along this warm/dry‐to‐cold/wet gradient, the proportion of facilitation links significantly increased at the cold/wet end (Figure 2c), whereas the proportion of facilitating and facilitated species significantly increased at the warm–dry end of the gradient (left hand side of Figure 2d, e), and the facilitation intensity received by the facilitated species significantly increased at the cold–wet end of the gradient (Figure 2f). These results indicate that at the cold–wet end of the gradient there are fewer facilitating species that are, at the same time, more generalist facilitators (i.e., each facilitating species facilitates a larger proportion of facilitated species) and also with larger positive effects on the abundance of the facilitated species.

### 3.3 Functional patterns of facilitation

**Figure 3:**
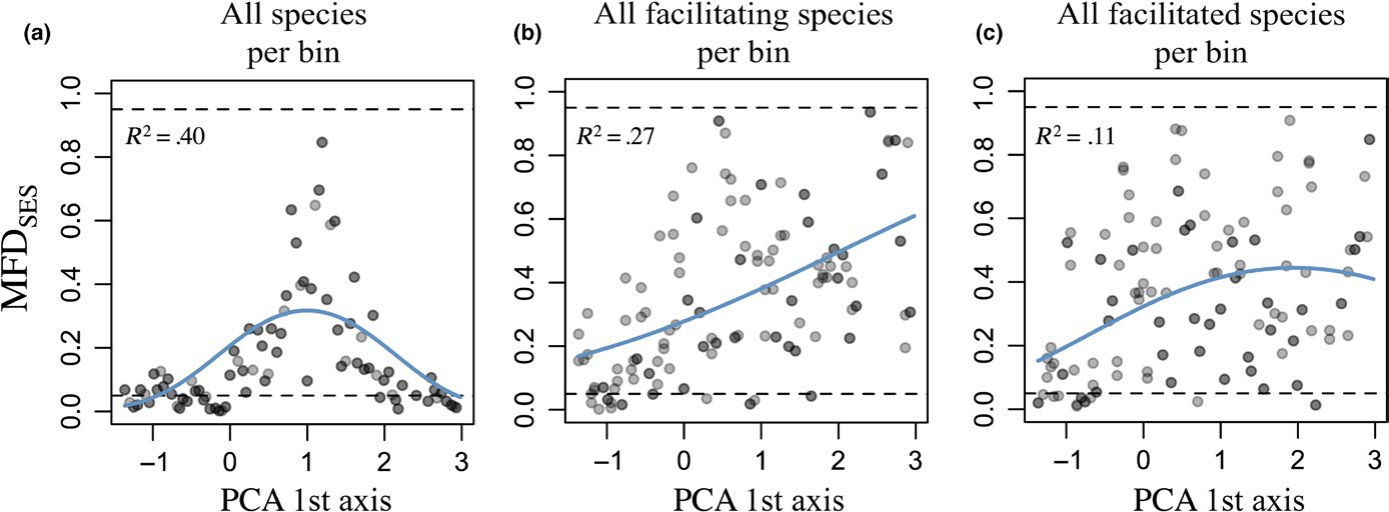
Functional distance among all species (a) and among facilitating (b) or facilitated species only (c) per bin. Each dot represents a bin, the horizontal dashed black lines indicate the significance thresholds for species detected to be more similar (.05) and more dissimilar (.95) than expected by chance. The blue lines indicate significant relationships between the similarity measures and the warm/dry‐to‐cold/wet gradient

The mean functional distance (MFD_SES_) between all species in a bin showed a significant Gaussian response along the environmental gradient (*p*‐val < .001, R^2^ = .40; Figure 3a). Facilitating species tended to be more similar among each other than expected by chance at the warm/dry edge, but this functional distance became more random at the cold/wet edge of the gradient (*p*‐val < .05, R^2^ = .27, Figure 3b). Facilitated species showed the same pattern (*p*‐val < .05, R^2^ = .11, Figure 3c), although less pronounced than among the facilitating species.

### 3.4 Species‐specific trends in facilitation intensity

**Figure 4:**
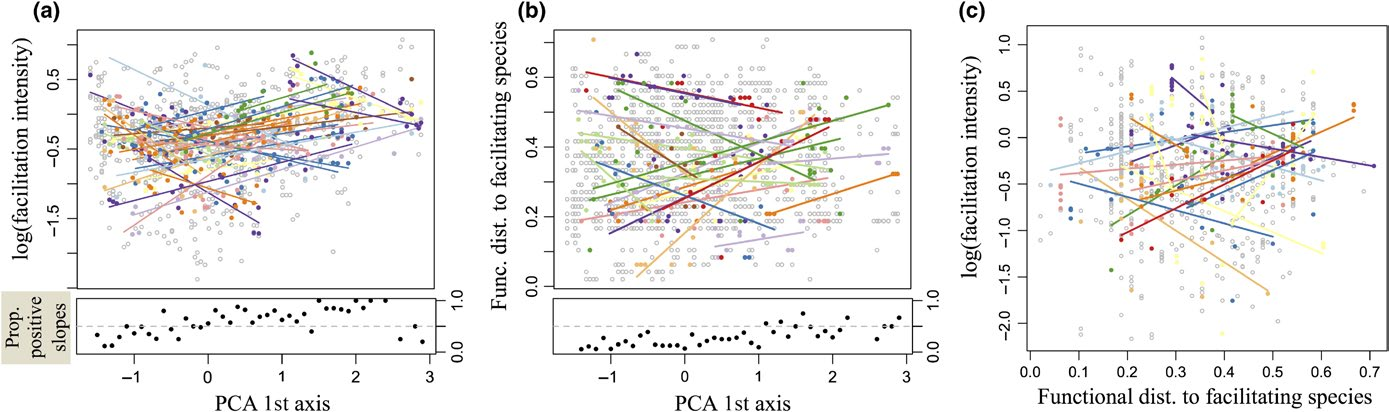
Functional similarity and facilitation intensity received by 46 facilitated species. (a) Species‐specific relationships between the log‐intensity of facilitation received and the environmental gradient. (b) Species‐specific relationship between the functional distance to the facilitating species and the environmental gradient. (c) Species‐specific relationship between the facilitation intensity and the functional distance to the facilitating species. Gray dots indicate all observations, while colored dots and lines indicate statistically significant relationships. The lower panels of (a) and (b) show the proportion of positive slopes among the significant relationships across the environmental gradient

When considering each facilitated species independently, 33 of 46 facilitated species (72%) showed significant trends in facilitation intensity received along the environmental gradient (19 positive and 14 negative trends; average R^2^ = .51; Figure 4a). For each facilitated species, we also tested whether the functional distance to its facilitating species changed along the gradient. Thereby, 22 of the 46 facilitated species (48%) showed significant trends along the environmental gradient (12 positive and 10 negative trends) with an average R^2^ = .37 (Figure 4b). Finally, considering each facilitated species independently, we found that 26 of 46 facilitated species (57%) showed significant relationships between their facilitation intensity received and their functional distance to their facilitators (15 positive and 11 negative relationships) with an average R^2^ = .25 (Figure 4c).

## 4 DISCUSSION

By introducing and validating a new analytical protocol for assessing facilitative interactions based on species co‐occurrence and co‐abundance patterns in community data, we are able to identify complex trends of facilitative interactions in species‐rich communities and along extended environmental gradients. First, in the case study of the Zermatt region, facilitation intensity was generally strongest at high elevation where species were exposed to cold (but not drought) stress, although these communities contained also fewer facilitating species than dryer/warmer communities. Second, the functional distance between facilitating and facilitated species changed along the stress gradient and seemed to influence the facilitation intensity but with no general trend across species. Below, we discuss our results and evaluate the strengths and weaknesses of this new method.

### 4.1 Facilitation patterns along an abiotic stress gradient

#### 4.1.1 General trends

In agreement with theoretical expectations, plant community richness and functional diversity were highest at intermediate elevation in the Zermatt region (Michalet et al., 2006). This indicates stronger environmental filtering and, thus, stronger abiotic stress in communities at both the warm/dry and the cold/wet edge of our steep gradient (ranging from an average moisture index of 8 mm to 136 mm of remaining, nonevaporated precipitation per month; Lavergne, Mouquet, Thuiller, & Ronce, 2010; Webb, Ackerly, McPeek, & Donoghue, 2002). Yet, the proportion of facilitating species was lowest at the cold/wet edge of the gradient (high elevations). This may be explained by the fact that facilitation under dry‐warm conditions—via desiccation protection through shading for instance—is frequent and has a limited cost for the species able to grow in very dry‐warm sites (e.g., Barbier, Couteron, Lefever, Deblauwe, & Lejeune, 2008; Maestre, Bautista, & Cortina, 2003; but see Maestre, Callaway, Valladares, & Lortie, 2009), whereas facilitation under cold condition—by sharing sparse nutrients or forming strong shelters for instance—comes at a greater cost, and thus, only few facilitating species may be able to provide it (e.g., cushion plants; Butterfield et al., 2013; but see Maestre et al., 2009).

Although the proportion of facilitating species was sparse at high elevations, it showed highest impact on the abundance of facilitated species (i.e., highest facilitation intensity). Our results are thus in line with previous findings, which stated that the intensity of facilitation is higher in cold environments (Callaway et al., 2002). But our results also demonstrate that facilitation is frequent in other types of stressful conditions (e.g., drought), although less intense there. Our findings provide hints about the complexity of facilitative interactions, where the number of facilitating species, the number of facilitated species per benefactor, and the intensity of facilitation vary along an extended environmental gradient.

#### 4.1.2 Facilitation intensity and functional distances are linked

To better understand the facilitation interactions along our stress gradients, we explored the functional relationship between facilitating and facilitated species. We found three major results. First, facilitating species significantly resembled each other at the warm/dry edge of our gradient, while they tended to be functionally different at the cold/wet edge of the gradient. This result suggests that facilitation at the cold/wet edge is mediated via a larger variety of processes (as provided by functionally more dissimilar species) compared to the warm/dry edge. Second, the functional distance between facilitating and facilitated species varied along the environmental gradient, but the direction of this change differed among facilitated species: Species present at the warm–dry end of the gradient showed on average negative trends, while species at the cold–wet end of the gradient showed on average mixed or positive trends (lower panel in Figure 4b). These results indicate that although species are generally more dissimilar at intermediate positions along the gradient (Figure 3a), pairs of facilitating and facilitated species tend instead to be more dissimilar at both ends of the gradient. Nonetheless, facilitation intensity received by the facilitated species (which generally increased toward the cold/wet edge of species ranges) did not appear to be directly linked to these changes in functional distance between the facilitated and facilitating species. These inconsistencies could indicate that facilitation is not emerging only from direct interactions, but probably also from indirect interactions of the facilitating species on the local biotic and abiotic environment. Such outcome overall recalls that not all species are necessarily stressed by the same environmental conditions and thus facilitated by the same mechanisms, even along one well‐known elevation gradient (Körner, 2003).

#### 4.1.3 Limitation of the methodology and perspectives

The new methodology proposed here is simple and has strengths and weaknesses. On the one hand, it allows for identifying broad patterns of facilitation without experimental manipulations of the system (i.e., avoiding the introduction of unnatural levels of abiotic stress to the system; Körner, 2003) and enables the integration of functional traits into the analyses of these patterns. On the other hand, it does not provide a detailed understanding of the actual processes driving facilitative interactions and thus cannot distinguish between direct and indirect facilitation mechanisms. Such a deeper understanding requires experimental manipulations or time series analyses. However, the method allows for screening potential facilitation patterns along large gradients using large datasets. Thereby, it provides a basis for developing hypotheses regarding underlying facilitation processes and designing specific experiments. Typically, it may be easier to pinpoint facilitation processes between species once we determine under which environmental conditions it occurs, and how its intensity changes along environmental gradients (e.g., if the facilitator provides a frost protection, facilitation should occur in cold conditions, and its importance should decrease with temperature). Our procedure is further useful for identifying the functional traits that characterize and are directly involved in the facilitative interaction for both the facilitating and the facilitated species.

It should be noted that we only grouped communities along one environmental gradient (i.e., moving window approach along the 1st PCA axis only). In this specific study system, the PCA axis used is in fact highly correlated with many other environmental variables, such as temperature, evapotranspiration, and moisture level (see Figure 2a). This is because our study system shows a very strong and dominating elevation gradient within a very small region (ca. 160 km^2^), which did not provide sufficient independence in moisture and temperature to study these gradients separately. However, in highly heterogeneous systems where the main environmental drivers are less or not correlated, it would certainly be necessary to instead group communities along two or more environmental axes. In such a case, an even larger database of community plots might be required to have sufficient material for statistical analyses available.

Another important point not yet analyzed in our framework is that, at the community scale, co‐occurring species can be at the same time facilitating (e.g., by modifying the local conditions) and competing with each other (e.g., by consuming the local resources). However, for predicting community dynamics, for example, under global change, quantifying the relative importance of competition and facilitation within communities is of utmost importance (McIntire & Fajardo, 2014). An analogous framework to the one presented here could be employed to identify and quantify competitive interactions (i.e., low co‐occurrence combined with a negative effect of competitors on their local abundances). Combining both frameworks would greatly enhance our understanding on how the balance of facilitation and competition varies with the intensity of abiotic stress experienced by the interacting species.

Overall, our method provides information useful for the refinement of coexistence theory from a functional perspective. Indeed, we have shown that along the studied environmental gradient some species tend to be facilitated by functionally dissimilar species and others by functionally similar species. This is in contrast to prevailing predictions in community ecology that functionally dissimilar species rather co‐occur due to competitive interactions, while similar species are expected to co‐occur due to environmental filtering (Weiher & Keddy, 1999). Therefore, our results call for caution when using only the functional distance between species as an indicator of the underlying coexistence mechanisms, as facilitation processes alone may favor the co‐occurrence of either similar or dissimilar species.

To conclude, we have introduced a simple and tractable method to identify and quantify facilitative interactions. Applying this method over a long moisture/temperature gradient in a species‐rich system revealed new evidence that facilitation, similarly to competition, can operate between functionally similar and dissimilar species, and that these differences can change along environmental gradients. Applying this approach to other systems (e.g., savanna, tropics, and forest) and biotic levels (e.g., birds, amphibians, and arthropods) will offer vast opportunities to identify the main stress gradients for different taxonomic groups and regions, and help better understand the facilitation mechanisms prevailing in different environments.

## CONFLICT OF INTEREST

None declared.

## AUTHOR CONTRIBUTION

LG, DZ, and NEZ developed the general ideas. NEZ collected the community dataset. LG performed all analyses. LG wrote a first draft of the manuscript, and all coauthors significantly contributed to improve it up to the final version.
