# Supplementary material for: Frequency and intensity of facilitation reveal opposing patterns along a stress gradient
Source: Ecol Evol. 2018 Jan 22;8(4):2171–81. doi: 10.1002/ece3.3855 (PMC5817155; doi:10.1002/ece3.3855)

**Appendix** **A. Material and method details**

*The simulations with VirtualCom*

As proof of concept that our approach is capable of reliably detecting facilitation and that it does not confound facilitation with other coexistence mechanisms, we used a virtual ecologist approach (Zurell et al. 2010), and simulated 4 different scenarios: facilitation, environmental filtering, competitive interactions, and neutral co-existence. To do so, co-occurrence patterns were generated using the recently published community assembly model *VirtualCom* (Münkemüller & Gallien 2015). *VirtualCom* has originally been developed to simulate community assembly under three possible processes, namely: environmental filtering, competitive interactions and neutral co-existence. Here, we extended it to include the option of simulating facilitative interactions

In brief, with *VirtualCom* the community assembly process starts with an initialization phase where individuals are randomly chosen from the species pool until the carrying capacity K is reached. Community composition then changes via asynchronous updating. In each time-step, K individuals are randomly selected and then replaced based on the parameterized assembly rules. The probability weight W*i,c* of an individual from species *i* to replace a random individual of community *c* is defined as:

$W_{i,c}={exp[ \beta}_{env}\times\log\left( P_{env,i,c} \right)+ \beta_{comp}\times\log\left( P_{comp,i,c} \right)+\beta_{facil}\times log(P_{facil,i,c})+ \log\left( {SP}_{abun}+\beta_{abun}\times P_{abun,i,c} \right)]$ (Eq. 1)

where $\beta_{env}$, $\beta_{comp}$, $\beta_{facil}$, and $\beta_{abun}$ weight the importance of environmental filtering, competition, facilitation and recruitment mechanisms. $P_{env,i,c}$, $P_{comp,i,c}$, $P_{facil,i,c}$, and $P_{abun,i,c}$ describe the probability of an individual of species *i*, to enter the community *c* according to environmental filtering, competition, facilitation and recruitment mechanisms (see Münkemüller & Gallien 2015 for more details on $P_{env,i,c}$, $P_{comp,i,c}$, and $P_{abun,i,c}$). The facilitation effect, $P_{facil,i,c}$, reflects a linear positive effect of the abundance of facilitating species *j* in community *c* (*N_j,c_*) for the facilitated species *i,* until the abundance of *i* reaches the abundance of *j*:

$P_{facil,i,c}={\{}_{1+N_{j,c} abundance of i < abundance of j}^{1 abundance of i \geq abundance of j}$ (Eq.2)

Equation 1 thus defines the relative importance of environmental vs. competition vs. facilitation vs. reproduction filters. Probability weights were then normalized to sum to 1 over all species in the species pool to obtain a probability of replacement for each species.

With this model we tested different scenarios of community assembly rules: environmental filtering ($\beta_{env}=\beta_{abun}=1$ and $\beta_{comp}=\beta_{facil}=0$), competition ($\beta_{comp}=\beta_{abun}=1$and $\beta_{env}=\beta_{facil}=0$), facilitation ($\beta_{facil}=\beta_{abun}=1$ and $\beta_{env}= \beta_{comp}=0$), and neutral ($\beta_{abun}=1$ and $\beta_{env}=\beta_{comp}=\beta_{facil}=0$).

Because we were specifically interested in the detection of facilitation mechanisms, we designed 4 specific facilitation scenarios where the facilitating species was facilitating either: 1, 2, 5, or 10 facilitated species. For each scenario (4 of facilitation + 3 of other mechanisms), we generated 50 different species pools containing each 50 species, where facilitating and facilitated species were chosen at random. From each of these species pool we assembled 50 communities (with a carrying capacity K= 200 individuals), together constituting one bin (i.e. one bin per species pool). In total we simulated 50 communities x 50 independent bins/species pools x 7 community assembly scenarios (17500 communities overall).

*The Zermatt dataset*

Within each community (figure A1), species abundances were recorded using the Braun-Blanquet cover scheme with 6 classes (1: less than 1%, 2: from 1 to 5%, 3: from 5 to 25%, 4: from 25 to 50%, 5: from 50 to 75%, 6: from 75% to 100%; Braun-Blanquet 1946), and then converted into relative abundances by: first replacing the cover classes by their mean percentages (0.5, 3, 15, 37.5, 62.5 and 87.5%), and then normalizing them to between 0 and 1 to obtain the relative abundance of each species.

*Mean functional distance between species*

MFD_SES_ is the quantile of the observed mean functional distance (MFD_obs_) vs. the mean functional distance obtained from communities generated by the null model (“null communities”; MFD_null_). In other words, MFD_SES_ is defined as the rank of the observed metric (MFD_obs_) within null communities (MFD_null_), divided by the number of null communities + 1 (here 999+1). Its score can thus vary between 0 and 1, where values superior to 0.5 indicate that species are more dissimilar than expected by chance (significantly so for MFD_SES_ > 0.95), and values inferior to 0.5 indicate that species are more similar than expected by chance (significantly so for MFD_SES_ < 0.05).

**Reference:**

Braun-Blanquet J. 1946. Über den Deckungswert der Arten in den Pfl anzengesellschaften der Ordnung Vaccinio-Piceetalia. *Jahresber. Naturforsch. Ges. Graubündens.* 130: 115–119.

Münkemüller, T., and L. Gallien. 2015. VirtualCom: A simulation model for eco-evolutionary community assembly and invasion. Methods in Ecology and Evolution 6:735–743.

Zurell, D., U. Berger, J. S. Cabral, F. Jeltsch, C. N. Meynard, T. Münkemüller, N. Nehrbass, et al. 2010. The virtual ecologist approach: simulating data and observers. Oikos 119:622–635.


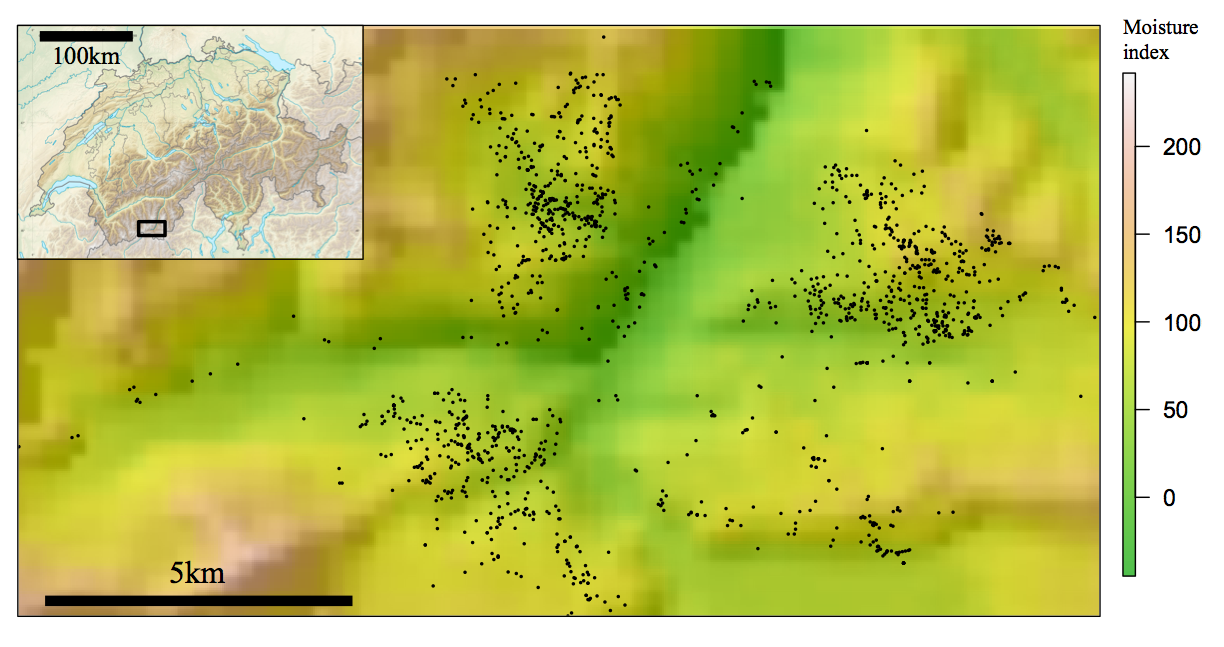


**Figure A1**. The Zermatt Mountain Region (Switzerland, in the top left inset) and the 1242 plant community relevés. The colour scale indicates the moisture index (yearly average of monthly moisture level; in mm x month^-1^) at 200m resolution, and the shading shows the orientation of the slopes.

**Appendix** **B. Additional results**

*Simulation error rates*

**Figure B1**. Error rates at detecting facilitation pairs obtained from process-based community assembly simulations. False positive error rates are indicated in orange and false negative error rates are indicated in blue. When environmental filtering, competition and neutral coexistence are simulated (first three panels on the left), all false positive error rates are null (orange dot). When facilitation is simulated (right panel), the mean false positive and mean false negative error rates are indicated with a solid line, while the standard deviation around this mean is represented with the shaded area around the mean. The classical 0.05 threshold value for acceptable error rate is indicated with a horizontal grey dash line.


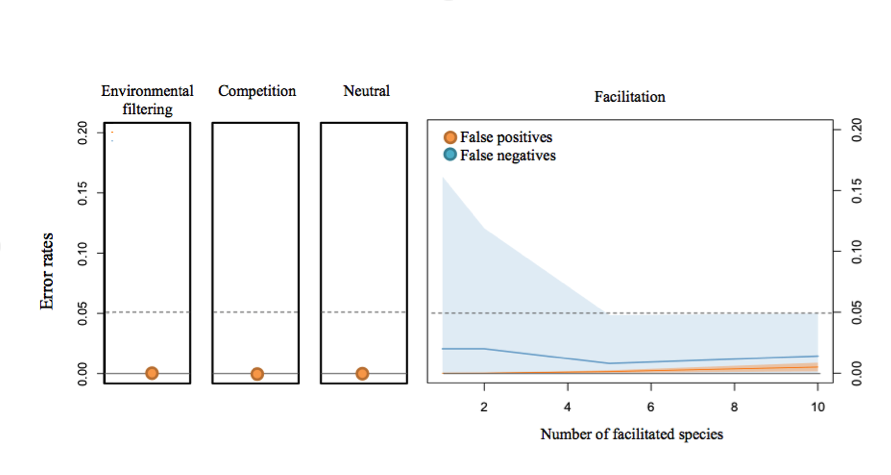

Supplement: Supplementary file 1 [file ECE3-8-2171-s001.docx]
